# Spiritual Needs, Religious Coping and Mental Wellbeing: A Cross-Sectional Study among Migrants and Refugees in Germany

**DOI:** 10.3390/ijerph19063415

**Published:** 2022-03-14

**Authors:** Kathrin Maier, Karol Konaszewski, Sebastian Binyamin Skalski, Arndt Büssing, Janusz Surzykiewicz

**Affiliations:** 1 Department of Educational Psychology in Social Work, Catholic University of Applied Sciences Munich, 80335 Munich, Germany; kathrin.maier@ksh-m.de; 2 Faculty of Education, University of Bialystok, 15328 Bialystok, Poland; k.konaszewski@uwb.edu.pl; 3 Institute of Psychology, Polish Academy of Sciences, 00378 Warsaw, Poland; sebastian.skalski@sd.psych.pan.pl; 4 Professorship Quality of Life, Spirituality and Coping, Faculty of Health, Witten/Herdecke University, 58455 Herdecke, Germany; arndt.buessing@uni-wh.de; 5 Faculty of Philosophy and Education, Katholische Universität Eichstätt-Ingolstadt, 85072 Eichstätt, Germany; 6 Faculty of Education, Cardinal Stefan Wyszynski University in Warsaw, 01938 Warsaw, Poland

**Keywords:** refugees, negative dysfunctional appraisal, spiritual needs, religious coping, wellbeing

## Abstract

It has been widely proven that resettlement is associated with negative psychological effects (e.g., increased depression and symptoms of post-traumatic stress disorder) among refugees. Therefore, there is an urgent need to improve the psychosocial functioning of migrants. This study assessed associations between negative dysfunctional appraisal (perceiving experiences as stressful), spiritual needs, religious coping and wellbeing. Data from paper-and-pencil questionnaires were collected from 744 refugees (69.8% male) aged 18–67 years (*M* = 27.99) with diverse backgrounds (including from Mashreq countries) who were resettled in Germany. Bootstrapping mediation analysis revealed that the relationship of dysfunctional appraisal and wellbeing among refugees is mediated by spiritual needs (i.e., existential and religious needs). Additionally, negative religious coping mediates the relationship between spiritual needs and wellbeing. The data obtained suggest the need for practitioners to focus on psychological interventions that strengthen spiritual needs in order to improve mental health among refugees.

## 1. Introduction

The number of people forcibly displaced from their homes is increasing, with data from the United Nations High Commission for Refugees (UNHCR) indicating that global forced displacement had exceeded 84 million by mid-2021 [1]. At the end of 2019, Germany had nearly 15 million refugees and migrants, as well as 309,000 asylum seekers, making it the largest host country for refugees and migrants in Europe. Half of the refugees came from Syria [2]. The scale of the phenomenon calls for research into psychosocial functioning among migrants. It is a challenge not only for researchers but also for psychologists and educators working in refugee centers in the field of psychological interventions and social policy of a given country, especially considering that leaving a country and resettlement are highly traumatic and stressful events. Numerous studies reveal that refugees have an increased vulnerability of mental health due to stressors prior to migration and the migration experience itself, as well as post-migration stressors such as separation from family members, social isolation, uprooting and experiences of discrimination [3,4]. Dysfunctional or negative appraisals of life events are linked to posttraumatic and depressive symptoms [5,6,7]. The perceived stress affects individuals and social functioning, which is conditioning their life situation in Germany [8,9,10,11,12,13].

Previous studies have indicated a high prevalence of post-traumatic stress disorder (PTSD) and depression in resettled refugee populations [14,15]. Fazel et al. [16], in a meta-analysis, showed a 10-fold higher prevalence of PTSD symptoms among refugees compared to general population data. Slewa-Younan et al. [15], in a review of Iraqi refugees resettled in Western countries, noted that full-blown PTSD was exhibited by 8 to 37.2% of participants, while depression ranged from 28.3 to 75%. Recovering from such stressful experiences may require a long recovery, as well as a concerted effort by researchers and practitioners to develop effective therapeutic interventions. In such a situation, studying acculturation strategies, refugees’ attitudes, coping styles and expectations for the future seems crucial for social service providers who assist these refugees.

Few data are available on spiritual needs and the use of religious coping, as well as their impact on wellbeing among refugees. On the other hand, the use of religion or spirituality to cope with trauma has been shown to be common during adaptation to traumatic events [17]. There is a growing body of literature showing that religion and spirituality might be an essential coping mechanism and support resource [18], especially for refugees [19,20,21]. In terms of psychosocial strategies, refugees utilize religion to endure challenges, losses and changes in life conditions. These could be complex and intersect with various individual, social and structural conditions [22,23]. Moreover, preliminary research findings suggest that religion and faith practices play an essential role in the mental health and integration of refugees in Germany, which provides insight into how mental health care can be provided in a religion-sensitive manner that offers alternatives to the social, cultural and linguistic barriers of the German health care system [24]. Ano and Vasconcelles [25] conducted a meta-analysis to integrate research on religious coping with adaptation to stressful events. They showed that religious coping methods produced positive psychological outcomes such as acceptance, hope, life satisfaction, optimism, spiritual growth and stress-related growth. Similar results were shown in meta-analyses [20,26,27,28,29,30]. Thus, in order to provide information on the potential role of spiritual needs and religious coping styles in adapting to life’s difficulties in the refugee group, in the present study, we collected data from different migrant groups living in Germany.

In this study, we address how spiritual needs and religious coping styles might contribute to the relationship between situation appraisal and mental wellbeing in refugees. Based on the assumption that medical and psychosocial care focuses mainly on the so-called ‘primary needs’ while neglecting the other needs, we focused on spiritual needs to meet refugees’ more comprehensive requirements. These so-called ‘secondary needs’ could be an important resource to cope with the new life situation in a foreign country and culture, as they utilize more complex resources to cope with the difficult situation [21,31,32]. Such a resource-oriented perspective seems to be even more relevant in the case of negative and/or dysfunctional appraisals of the new post-migration life situation. Numerous studies reveal that refugees have an increased vulnerability of mental health due to stressors prior to migration, the migration experience itself as well as post-migration stressors such as separation from family members, social isolation, uprooting and experiences of discrimination [3,4]. As such, a dysfunctional interpretation of the current life situation as a punishment or threat can be understood as an additional risk factor concerning the mental wellbeing of refugees. Following the transactional model of stress and coping [33], the appraisal of a situation as a threat to one’s own wellbeing motivates coping behavior in an attempt to deal with stressful demands and uphold or restore a positive psychological state. However, actual coping behavior depends on a person’s evaluation of his or her resources and options for coping. With reference to Emmons’s cognitive-motivational conceptualization of spirituality as a form of intelligence to enable problem-solving and goal attainment [34], the conscious awareness of spiritual needs might function as a motivational force to initiate coping activities. This directly leads to Pargament’s theory of religious coping as a complementary form of support ‘when other sources of support are lacking’ [35]. As such, religious coping ‘complements nonreligious coping by offering responses to the limits of our personal powers’ [35]. Religious coping, in turn, relates to mental health and wellbeing [36,37,38,39]. 

**Figure 1:**
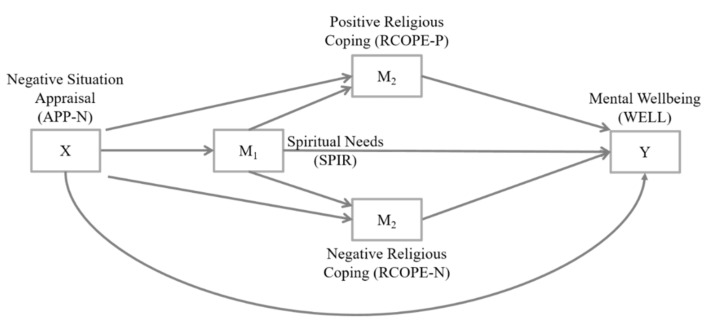
Conceptual form of the expected mediation model.

Based on these theoretical approaches, this study proposed a conceptual model of the relationship between negative dysfunctional situation appraisal and mental health. As depicted in Figure 1, the following hypotheses were derived. (1) Based on previous findings on the link between negative appraisals of life events and mental symptoms or disorders, it was assumed that a negative situation appraisal (X) of refugees reduces their mental wellbeing (Y). (2) Referring to Lazarus’s transactional model of stress and coping and Pargament’s theory of religious coping, an indirect association between a negative situation appraisal (X) and mental wellbeing (Y) was also expected through religious coping activities. With respect to the distinction between positive and negative religious coping methods [40], a parallel mediator model was hypothesized. Based on former findings on the association between religious coping style and mental wellbeing, positive religious coping (M2) was expected to reveal a positive mediating relationship on mental wellbeing, while negative religious coping (M3) was expected to imply a negative mediating relationship. (3) With respect to Emmons’s cognitive-motivational conceptualization of spirituality, an indirect association between a negative situation appraisal (X) and mental wellbeing (Y) through spiritual needs (M1) was also assumed. However, spiritual needs are not necessarily the result of low spiritual wellbeing or low life satisfaction; these associations can be observed depending on the course and activity of the disease, but also as a result of various other complex interacting factors (overview in [41]). In patients with chronic diseases, spiritual needs are usually positively related to positive interpretations of the current life situation, but not relevantly related to negative appraisals (overview in [41]). (4) Combining the theoretical approaches into an overall model, a mediation model with serial and parallel mediation properties was additionally hypothesized. As such, the influence of a negative situation appraisal (X) on mental wellbeing (Y) would be sequentially mediated through a serial mediation path with spiritual needs (M1) as a first and religious coping as a second factor, whereby positive religious coping (M2) and negative religious coping (M3) were conceptualized as parallel mediator variables in the serial mediation model. 

## 2. Materials and Methods

### 2.1. Participants and Procedure

The participants in this cross-sectional study were 744 refugees in Germany coming from different countries; 519 were males (69.8%) and 188 females (25.3%), while 37 participants did not report their gender. The mean age of the sample was *M* = 27.99 years, ranging from 14 to 67 years (*SD* = 8.50). Demographic characteristics of the sample are detailed in Table 1.

The present study was conducted in Germany between 2018 and 2020 with the approval of the Ethics Committee of the Institute of Psychology, Polish Academy of Science (#11/03/18). Recruitment was conditional on having refugee status. Participation in the study did not entail additional recruitment criteria. The study was anonymous and voluntary. Data from paper-and-pencil surveys were collected through collaboration with refugee centers in Bavaria. The sampling took place in Bavaria, especially in the regions around Munich and Ingolstadt. The refugee population can be understood as a hard-to-reach population. Thus, a regular sampling procedure such as snowball-sampling based on a random sample of people from a given population was not possible. Instead, respondent-driven sampling was used, which started with a non-random convenience sample of people who then selected other people from the refugee population and so on. The convenience sample was based on key people in refugee institutions, refugee centers and refugee camps who were asked to distribute the questionnaire among their clients and to active other relevant and accessible networks. With respect to the hard-to-reach population, the survey was a paper-based questionnaire only which took approximately 20 min to complete. The questionnaires were offered in English, Arabic, Farsi, German and French.

### 2.2. Measures

Wellbeing (WELL). Mental wellbeing was measured as a dependent variable using the five-item World Health Organization Wellbeing Index (WHO-5) [42]. The instrument is a short version of the 10-item scale (WHO-10) [43], based on originally 28 items that were basically derived from the Zung scales for depression, distress and anxiety [44]. The WHO-5 is a unidimensional questionnaire measuring positive wellbeing synonymous with mental health during the last 14 days. All items of the WHO-5 are phrased positively with a six-point response format ranging from 0 (none of the time) to 5 (all of the time). The scale has demonstrated high clinometric validity and can be used across a wide range of study fields [45]. In the present study, Cronbach’s alpha was 0.89. The participants rate each of them on a 6-point scale, from 1 = “at no time” to 6 = “all of the time”.

Spiritual Needs (SPIR). Spiritual needs were conceptualized using the Spiritual Needs Questionnaire (SpNQ) [46,47]. The instrument measures psychosocial, existential and spiritual needs, referring to four core dimensions of spirituality, namely connection, peace, meaning/purpose and transcendence [48]. Based on the assumption that refugees suffer from high levels of stress, the instrument seemed suitable for this sample. The instrument contains 20 main items and additional (“informative”) items that are coded on a four-point scale from disagreement to agreement, ranging from 0 (not at all) to 3 (very strong). In the original scale validation, the items are grouped into four dimensions of spiritual needs with Cronbach’s alpha ranging from 0.71 to 0.92. These subscales were also applied for the present study: Religious needs (SPIR-REL), i.e., the need to pray or to read spiritual/religious books (*α* = 0.80); Existential needs (SPIR-EXIST), i.e., the need to find meaning in illness and/or suffering or to reflect upon one’s previous life (α = 0.83); Needs for inner peace (SPIR-PEACE), i.e., the need to find inner peace or to plunge into the beauty of nature (*α* = 0.78); giving/generative needs (SPIR-GEN), i.e., the need to give solace to someone or to feel connected with family (*α* = 0.78). SPIR is characterized by good psychometric properties (Cronbach’s α = 0.92). The response format varied from 1 (not at all) to 4 (very strong). The average response score was calculated.

Religious Coping (RCOPE). Religious coping was measured using the Brief Measure of Religious Coping (Brief RCOPE) by Pargament et al. [49,50]. The 14 items of the instrument are divided into two subscales, each consisting of seven items. The first subscale identifies positive religious coping methods, including a sense of connectedness with a transcendent force, a caring image of God and a secure relationship with God. Cronbach’s alpha for this subscale was ranging from 0.67 to 0.94 with a median alpha of 0.92. The second subscale focuses on negative religious coping methods, based on signs of spiritual tension, conflict and struggle with God. Cronbach’s alpha for this subscale was between 0.60 to 0.90 with a median alpha of 0.81 [50]. Items are measured on a four-point Likert scale ranging from 0 (not at all) to 3 (a great deal) to indicate the extent to which an individual uses specific methods of religious coping when facing critical live events. In this study, Cronbach’s alpha for the positive religious coping subscale (RCOPE-P) was 0.86 and 0.91 for the negative religious coping subscale (RCOPE-N). Responses were added to a mean score. 

Negative Appraisal of Life Situation (APP-N). The tendency to negatively evaluate the current life situation was measured using three items of the Illness Interpretation Questionnaire (IIQ) [51]. The measure refers to the cognitive evaluation of the situation according to Lazarus and Folkman theory [33]. According to the selected statements, the experienced situation can be considered as: (a) punishment, (b) an adverse interruption of life and (c) a threat/enemy, which initiates the existence of a stress relationship. All items are scored on a five-point scale from disagreement (0: does not apply at all) to agreement (4: applies very much). In this study, exploratory factor analysis revealed a univariate structure for the statements used. The factor created was named ‘negative dysfunctional judgment’ and had a Cronbach’s alpha of 0.82, indicating satisfactory internal consistency of the scale.

### 2.3. Data Analysis

A preliminary examination of the variables was performed. Specifically, the Kolmogorov–Smirnov test was used to assess normality, while Levene’s test was used to assess homoscedasticity. The results of this examination support the application of the parametric tests that were applied in this study. Pearson’s *r* correlation analysis and regression analysis were used to determine the relations between the variables. The mediation model was assessed using Hayes’ Process macro. The significance level was determined at *p*  <  0.050. The effect size was assessed based on *R^2^*. Data analysis was conducted in IBM SPSS Statistics 26 (International Business Machines Corporation, New York, The United States of America). Since we used different language versions of the questionnaires in the study, and many of these versions have not yet been validated for psychometric properties, we first conducted a confirmatory factor analysis of all the tools, separately, for each language version. Saturation of statements with particular factors was arranged according to theoretical assumptions of the scales. Each time, we obtained RMSEA < 0.08 and GFI > 0.90, which allowed us to validate the structure of the scales (assessed in SPSS Amos 27).

## 3. Results

The means, standard deviations, standard errors and correlations among the study variables are presented in Table 2. In the sample, mean scores of mental wellbeing were moderate (*M* = 3.59, *SD* = 1.09). All spiritual needs scored were rather high. Lowest (among high) mean scores were found for existential needs (*M*_Existential_ = 2.41, *SD* = 0.78), indicating a strength between ‘somewhat’ and ‘strong’. All other categories of spiritual needs were expressed as ‘strong’. Participants predominantly indicated the use of positive religious coping strategies (*M* = 2.84, *SD* = 0.77), while negative religious coping was reported to be used ‘somewhat’ (*M* = 1.71, *SD* = 0.79; *t*_(743)_ = 28.39, *p* < 0.001). The mean tendency towards negative situation appraisal was low (*M* = 1.98, *SD* = 0.97). In this study, neither age, gender, asylum status nor any of the other sociodemographic factors affected the results in a statistically significant way. 

**Figure 2:**
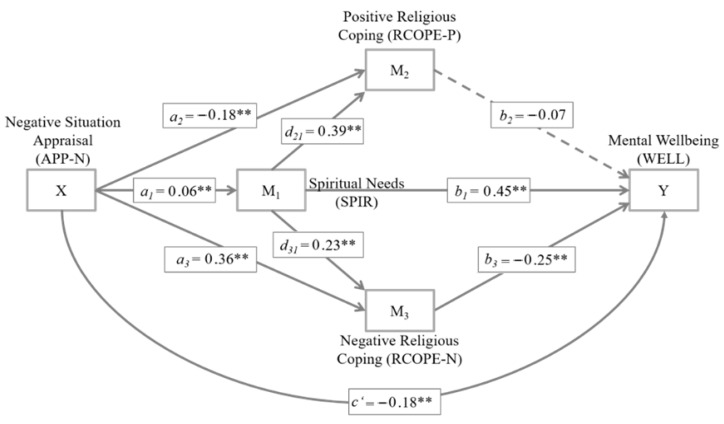
Mediation model with serial and parallel mediation properties to predict mental wellbeing in refugees. Model coefficients are reported in unstandardized form. Solid lines indicate significant paths, dashed lines non-significant paths. The a paths indicate relations between the predictor and the mediators; the b paths indicate relations between the mediators and the criterion; the c’ path indicates the relation between the predictor and the criterion when controlled for the mediators; the d paths indicate the relation between the mediators. ** p < 0.010.

Figure 2 illustrates the mediation model. The predictor (X) of the hypothesized mediation model was the extent of negative situation appraisal (APP-N). The first mediator (M_1_) was the total score of spiritual needs (SPIR). M_1_ was conceptualized as a serial mediator with a pathway of influence to the two other mediators M_2_ and, namely positive (M_2_: RCOPE-P) and negative religious coping styles (M_3_: RCOPE-N). M_2_ and M_3_ were conceptualized as parallel mediator variables in the serial mediation model. The outcome variable (Y) was mental wellbeing (WELL). Model coefficients are reported in unstandardized form to allow a direct scale-bound data interpretation.

Table 3 summarizes the model coefficients and reports the effect sizes (adjusted *R*^2^) for the four multiple regression analyses to estimate the coefficients of the mediation model. Significance tests of indirect, direct and total mediation effects are displayed in Table 4. The effect sizes for these effects are provided using the completely standardized effect, which is equivalent to the standardized regression coefficient *β*. As such, it is invariant to linear transformations of X, M or Y and has an intuitive interpretation [52].

The specific indirect effect through the first mediator M_1_ (*B*_specific M1_ = 0.028, *SE*_specific M1_ = 0.011, 95%*CI* (0.008,0.053)) was significant, indicating that refugees with higher in negative situation appraisal (APP-N) scores reported more spiritual needs (SPIR) than refugees who stated lower levels of negative situation appraisal (*a*_1_ = 0.06, *p* < 0.010). More spiritual needs (SPIR) subsequently related to better mental wellbeing (WELL; *b*_2_ = 0.45, *p* < 0.001). In addition, there was a specific indirect effect through the third mediator M_3_ (*B*_specific M3_ = −0.089, *SE*_specific M3_ = 0.023, 95%*CI* (−0.044, −0.134)), indicating that a stronger tendency towards negative situation appraisal (APP-N) also exerted more negative religious coping (RCOPE-N; *a*_3_ = 0.36, *p* < 0.001), which was subsequently associated with poor mental wellbeing (WELL; *b*_3_ = −0.25, *p* < 0.001). In contrast, there was no specific indirect effect through the second mediator M_2_ (*B*_specific M2_ = 0.013, *SE*_specific M2_ = 0.010, 95%*CI* (−0.007,0.035)), as positive religious coping (RCOPE-P) did not exert a significant effect on mental wellbeing (WELL; *b*_2_ = −0.07, *p* = 0.164). Nevertheless, refugees who score higher on negative situation appraisal (APP-N) reported less positive religious coping activities compared to refugees who stronger negated a negative situation appraisal (RCOPE-P; *a*_2_ = −0.18, *p* < 0.001). The specific indirect mediation path through M_1_ and M_3_ was just significant (*B*_specific M1–M3_ = −0.004, *SE*_specific M1–M3_ = 0.002, 95%*CI* (−0.001, −0.008)). This serial mediation path indicated a relationship between negative situation appraisal (APP-N) and a greater extent of spiritual needs (SPIR; *a*_1_ = 0.06, *p* < 0.010), which subsequently related to more negative religious coping activities (RCOPE-N; *d*_31_ = 0.23, *p* < 0.001). These in turn implied lower mental wellbeing (WELL; *b*_3_ = −0.25, *p* < 0.001). The specific indirect serial mediation path through M_1_ and M_2_ did not reach significance (*B*_specific M1–M2_ = −0.002, *SE*_specific M1–M2_ = 0.002, 95%*CI* (−0.006,0.001)). While a negative situation appraisal (APP-N) was linked to higher spiritual needs (SPIR; *a*_1_ = 0.06, *p* < 0.010), which subsequently related to more positive religious coping (RCOPE-P; *d*_21_ = 0.39, *p* < 0.001), positive religious coping was not associated with mental wellbeing (WELL; *b*_2_ = −0.07, *p* = 0.164). Independent of the effects of the proposed mediators, there was also a significant direct effect of negative situation appraisal (APP-N) and mental wellbeing (WELL), indicating lower mental wellbeing in the case of a more negative situation appraisal (*c’* = −0.18, *SE*_direct_ = 0.046, *p* < 0.001). Yet summing up indirect and direct influences, the total effect of negative situation appraisal on mental wellbeing did not exert significance (*B*_total_ = −0.044, *SE*_total_ = 0.041, *p* = 0.289). 

To gain a deeper understanding of the role of spiritual needs, additional analyses were carried out. In addition to the total score of spiritual needs as first mediator (M1: SPIR) in the hypothesized mediation model, the model was also analyzed separately for each of the four addressed dimension of spiritual needs. The results for the specific dimensions of existential spiritual needs (SPIR-EXIST) and religious spiritual needs (SPIR-REL) were in full accordance with the findings for the total score of spiritual needs presented above. The dimension of generative needs (SPIR-GEN) in contrast revealed different findings. There was no specific indirect effect of negative situation appraisal on mental wellbeing through generative needs (*B*_specific M1_ = −0.003, *SE*_specific M1_ = 0.001, 95%*CI* (−0.019, 0.010)), as negative situation appraisal did not relate to this dimension of spiritual needs (*a*_1_ = −0.01, *p* = 0.618). As a consequence, the specific indirect serial effects of negative situation appraisal on mental wellbeing through generative needs (M1) and positive religious coping (M2, *B*_specific M1–M2_ = 0.0001, *SE*_specific M1–M2_ = 0.0006, 95%*CI* (−0.001, 0.001)), and through generative needs (M1) and negative religious coping (M3), respectively, were not significant (*B*_specific M1–M3_ = 0.0003, *SE*_specific M1–M3_ = 0.0009, 95%*CI* (−0.002, 0.001)). Generative needs themselves in contrast related positively to mental wellbeing (*b*_1_ = 0.26, *p* < 0.001), positive religious coping (*d*_21_ = 0.30, *p* < 0.001) and negative religious coping (*d*_31_ = 0.10, *p* < 0.050). Regarding the specific indirect mediation effects of negative situation appraisal on mental wellbeing through religious coping activities, findings were in line with the above presented results based on the total score of spiritual needs. The results for the dimension of needs for inner peace (SPIR-PEACE) also differed from the findings based on the total score of spiritual needs. First, the specific indirect effect of negative situation appraisal on mental wellbeing through needs for inner peace was not significant (*B*_specific M1_ = −0.013, *SE*_specific M1_ = 0.008, 95%*CI* (−0.031, 0.001)). Second, the specific indirect serial effect through needs for inner peace (M1) and negative religious coping (M3) was significant, as expected (*B*_specific M1–M3_ = −0.003, *SE*_specific M1–M3_ = 0.002, 95%*CI* (−0.0004, −0.006)), yet the directions of the underlying relationships followed a different pattern: a greater extent of negative situation appraisal implied lower spiritual needs for inner peace (*a*_1_ = −0.09, *p* < 0.001), which in turn were linked to higher negative religious coping. Higher levels of negative religious coping subsequently related to poor mental wellbeing (*b*_3_ = −0.31, *p* < 0.001). All other results, i.e., the non-significant specific indirect serial effect through needs for inner peace and positive religious coping, as well as the specific indirect effects through religious coping activities only, were fully in line with the above presented findings. 

## 4. Discussion

The focus of this cross-sectional study was to address how a negative and dysfunctional appraisal of the current life situation and mental wellbeing are linked among refugees in Germany. Based on multiple theories, a mediation model with serial and parallel mediation properties was proposed, including spiritual needs and religious coping methods as key variables in the relation between predictor and criterion. Overall, the depicted mediated relationships were supported. 

Refugees who indicated a more dysfunctional-negative situation appraisal of their current life situation were more likely to experience poor mental wellbeing. Findings are in line with several studies suggesting a link between negative appraisals of life events (i.e., perceived stress or stress appraisal) and mental symptoms. This accounts for example in the context of epidemics or pandemics [53], cumulative life adversities in traumatized communities [54], life-threatening illness in terms of advanced cancer [55], acculturative stress [56], as well as regarding daily life stressors in the general population [57].

Findings of the present study revealed a link between negative appraisal of refugees’ life situation and increased negative religious coping, which in turn was associated with lower mental wellbeing. On the other hand, negative situation appraisal was associated with less positive religious coping which in turn did not relate to mental wellbeing. Thus, the assumed association chain of negative situation appraisal, religious coping and mental wellbeing could be maintained for negative religious coping only. These findings are in line with previous results indicating mediating [58] or moderating effects of negative religious coping on the relationship between stress appraisal (i.e., negative appraisal of a life event) and variables of psychological distress [59]. Throughout the literature, negative religious coping is associated with higher levels of psychological symptoms such as depression, anxiety and maladaptive emotion regulation [60,61,62,63]. This negative link between negative religious coping and mental wellbeing was also revealed in our study. Moreover, the non-significant mediation path in case of positive religious coping is in line with previous research. Ambiguous findings exist on the association between positive religious coping and mental wellbeing. While some studies have indicated better mental wellbeing and fewer psychological symptoms in the case of positive religious coping [38,62], the majority of studies revealed no respective relationship [63,64,65,66,67]. Overall, positive religious coping has been shown to have less impact on mental health outcomes [61,63]. This could be explained by the fact that the coping strategies are used to cope with the stressor, but this will not guarantee the expected outcome, especially depending on the significance and duration of the stressor. With respect to the situation of the refugees, they are still living in the refugee camp; only half of them know that they will get asylum, and all nevertheless suffer from the experiences of escape from their home. In accordance with these findings, positive religious coping has shown only marginal buffer effects in moderated or mediated relationships between stress appraisal and mental wellbeing [68], or no buffer effects at all [58,59]. It is important to point out that the missing buffer effect of positive religious coping in our study not only relates to the missing relationship between positive religious coping and mental wellbeing. In addition, the stress appraisal variable—the dysfunctional situation interpretation as a punishment or threat—did not motivate positive religious coping techniques but was rather linked to less positive religious coping activities. Thus, thinking of religious dimensions in the face of a perceived ‘punishment’ on a no-fault basis (i.e., the post-migration situation) seems to directly activate external attribution processes to a punishing God instead of positive images of God. It should be noted that according to Lazarus and Folkman’s theory [33], punishment understood in this way concerns the feeling of harm that has occurred, as a consequence of which the individual loses valued resources and/or objects, which may be accompanied by anger, grief, sadness, harm and external locus of control—e.g., the belief that the cause of events is due to the activity of God.

A negative situation appraisal was linked to more spiritual needs (particularly existential and religious needs), which in turn related to better mental wellbeing. These findings are in line with Pargament and Brant [69], who emphasized the meaning of spirituality and religion, especially in demanding or difficult situations where other solutions are lacking. The conscious awareness of one’s spiritual needs might open up new perspectives and strength. As such, spirituality and spiritual needs function as an important resource in the face of adversities [48]. Previous findings in fact revealed an association between spiritual needs and higher quality of life [70], as well as better mental wellbeing or less psychological stress, respectively, especially in threatening situations such as chronic illness [71,72,73]. Our results are in line with these findings, as all four dimensions of spiritual needs correspondingly related to mental wellbeing. Further analyses into the different dimensions of spiritual needs however revealed that their mediating effect on the relationship between negative situation appraisal and mental wellbeing only applied to existential and religious needs, not to inner peace needs of generativity needs. A possible explanation of these findings might point back to the dysfunctional appraisal of the current life situation as a punishment or threat. This view seems to foster helplessness and hopelessness, as underlying causal attributions of this situation interpretation are external and stable. Thus, searching for meaning can be understood as an important process of (self-)acceptance in moments of crisis, which is necessary to adapt to the situation and to finally perceive growth [74,75]. Similarly, religious activities like praying, reading spiritual books or attending religious ceremonies can be viewed as a way to cope with the adverse situation 41 or to find comfort [76] and to uphold a sense of active involvement and proactive action even in the face of hopelessness and threat [77,78]. The need for inner peace, in contrast, focuses more on forgiveness, resulting in states of inner peace, in hope and oneness with nature as a desired source of (‘paradisiac’) light-heartedness. As such, these seem to be opposite and incompatible with the dysfunctional appraisal of the post-migration situation as a punishment and threat. Thus, a negative association between the need for inner peace and the dysfunctional appraisal was found in the present study’s data. Generative needs, which focus on generative connectedness with significant others (mainly the direct family which is in most cases absent), did not relate to dysfunctional situation appraisal at all. Facing hopeless and helplessness, the presence of family members and friends might be a source of support, yet does not seem to enable a specific perspective of dealing with the adversity of the situation. It is also important to note that the negative assessment of the situation does not apply to all refugees. Certainly, according to some researchers, settling in Germany may seem to foster the opposite of helplessness and hopelessness compared to remaining confined to a home country torn by war with no view of any imminent end due to the involvement of international actors using the Mashreq and Africa as geopolitical playgrounds [28,29,30].

A serial mediation path with spiritual needs as a first and religious coping as a second factor could be revealed, yet for negative religious coping only. Refugees with a negative appraisal of their current situation were more likely to experience spiritual needs. In turn, higher spiritual needs were associated both with more positive as well as negative religious coping. However, only negative religious coping was associated with mental wellbeing, indicating poorer mental wellbeing in case of more negative religious coping. These results follow the preceding theoretical framework on Emmons’s motivational conceptualization of spirituality [34] and previous findings on spirituality as a resource to cope. As such, spirituality or spiritual needs respectively seem to be a resource to motivate nonreligious coping [79,80] as well as religious coping [48,81]. Focusing on religious coping, both positive and negative coping methods were activated through spiritual needs. This is also in line with previous findings, indicating that positive and negative religious coping styles are not mutually exclusive in a person, but might be both present at the same time [37,60,82,83]. Facing threatening situations in life, people seem to predominantly rely on positive religious coping, while negative religious coping is used only to a limited degree [37,60,84]. This also applies in the present study. Refugees not only reported more positive compared to negative religious coping in total, but spiritual needs also related stronger to positive than to negative religious coping techniques. Those findings are in line with the conceptual models, as spiritual needs may indicate a desired resource rather than a dysfunctional behavior [41]. The next step of the proposed serial mediation model was the link between religious coping and mental wellbeing, which was already discussed in hypothesis 2. The above reported results also explain why the proposed serial mediation path was finally non-significant for positive religious coping. 

## 5. Practical Implications and Recommendations

Considering the practical implications of our findings, it is of great importance to provide adequate support to refugees and to be aware of their spiritual needs. The activation of spiritual needs was shown to be an important resource to buffer the negative effects of a dysfunctional situation appraisal, which otherwise is directly linked to worse mental wellbeing, as well as negative religious coping. Among people with chronic diseases, spiritual needs are stronger related to positive appraisals than to negative interpretations [47]. This buffering effect of considering spiritual needs lies in their potential to activate positive beside negative religious coping techniques. However, this will not guarantee wellbeing as an outcome, particularly when the life situation still is difficult and the stressors are persisting. Even though positive religious coping was not directly associated with better mental wellbeing as a subsequent function, numerous studies have revealed correlative associations with positive psychological domains like hope [67,85,86], stress-related growth [66,87], higher self-esteem [88,89], positive affect [90] and mindfulness [89]. As such, positive religious coping as a process seems to enhance awareness of available personal resources, which may strengthen resilience against mental illness. Referring to these psychological benefits, the findings in addition reveal that positive religious coping mitigated the negative impact of religious or spiritual struggles in terms of negative religious coping on mental health [37]. Referring to the dysfunctional influence of negative religious coping on mental wellbeing as well as the importance of spiritual needs to interrupt a negative dynamic between dysfunctional appraisal and mental health issues, an inclusion of spiritual and religious issues and addressing spiritual needs into counselling as well as psychological treatment would be of great importance. Clients should be actively encouraged to express their moral and religious values, their needs, hopes and expectations. These in turn not only reveal possibly dysfunctional cognitive and emotional styles, such as predominantly negative religious coping, but also offer a basis for the client to develop acceptance and self-empathy. This in turn is a fundamental therapeutic requirement to enable changes where changes are possible (i.e., changing dysfunctional appraisal styles) and adaptation where circumstances cannot be changed (i.e., losses due to migration). In case of a predominantly negative religious coping style, therapeutic interventions can be offered to encourage the client to reduce negative and maximize positive religious coping techniques instead. Acceptance and Commitment Therapy, a ‘third-wave’ cognitive-behavioral therapy, offers interesting perspectives in this context, as suggested by Karekla and Constantinou [83]. Spiritual needs and religious coping are important variables in relation to the ability to cope with a traumatic situation and are an area that is currently receiving increasing attention in the psychological and psychotherapeutic community. As these fields evolve and newer research findings on coping mechanisms in the area of psychological well-being emerge, it is important that psychologists can incorporate research findings into their clinical practice. The goal should be to recognize the assessment of life situation and the diagnosis of spiritual needs, which will contribute to more effective and efficient therapies, especially in the case of people subjected to strong stress factors, such as, for example, refugees. New therapeutic approaches, such as acceptance and commitment therapy, are very promising in this field and should be further used [83].

## 6. Limitations and Future Studies

These data contribute to our understanding of the relationship between dysfunctional judgment, coping, spiritual needs and wellbeing among refugees. Despite their strengths, the results come with some limitations that must be considered before broad generalizations can be made. The cross-sectional nature of the study does not allow for conclusive judgments about cause and effect. Second, the study did not control for refugees’ experience of other traumatic events beyond resettlement, which could exacerbate the assessed phenomena. Cultural diversity of participants was further not included as a co-factor. The mean tendency towards negative situation appraisal was low (*M* = 1.98, *SD* = 0.97). In this study, neither age, gender, asylum status nor any of the other sociodemographic factors affected the results in a statistically significant way. Third, over 20% of participants did not declare their asylum status. Although the above has a marginal effect on the evaluation of the associations between psychological variables, it is subject to some error to conclude that there are no significant differences in the severity of these variables due to refugee status. On the other hand, our findings in this regard correspond with the consensus in the literature. In future research, it seems interesting to use experimental techniques (e.g., manipulation of the level of spiritual needs), as well as to consider the social support received, which may moderate the relationship between dysfunctional appraisal and wellbeing. Future studies should also include health service variables, such as satisfaction with care and quality of relationship, which \ could be related to refugees’ mental wellbeing. Moreover, the persistence of mental wellbeing over time needs to be examined.

## 7. Conclusions

Previous studies have indicated that experiencing displacement can be a highly stressful and traumatic phenomenon. The present study was designed to assess psychological resources related to spirituality that may improve functioning among refugees. We were among the first to show that the relationship between a dysfunctional appraisal (perceiving experiences as stressful) and wellbeing among refugees can be explained by spiritual needs. Additionally, negative religious coping may mediate the associations between these needs and wellbeing. The findings point out that religion and spirituality play a significant role in mental health and so show an important impact on integration of refugees in Germany, by raising awareness that not only culturally sensitive work with refugees as a basis of communication but also their health resources are co-determined by religion and spirituality. In consultancy contexts, the results could be helpful to generate support for health care providers and refugees who may be reluctant to discuss mental health issues due to shame to surpass religious privacy or other matters. It is a useful understanding of preferred ways of understanding spiritual needs and coping with severe problems among immigrants [30,91,92]. In therapeutic work, however, it is important to first identify refugees’ actual goals for their stay in the host country. It may be that some will accept bearing the costs and experiencing the trauma in order to improve their living conditions. Such individuals may benefit less from refugee integration or not be interested in it at all. Nevertheless, by using that kind of spiritual assessment, counselling can assess patients for unmet religious and spiritual needs, coping methods and can use interventions to deepen meaning-making within the counsellor–client relationship [24,93]. While health professionals can effectively identify the religious and spiritual needs of refugees, meeting these needs may require the involvement of priests and imams of the relevant religious denominations. In our view, the integration of refugees in Germany may not pose an obstacle to the maintenance and/or development of religious or spiritual resources due to the high level of religious tolerance and diversity in Germany.

## Figures and Tables

**Table 1:** Further demographic characteristics of the sample.

Variable	*n*	%
Educational status		
Illiterate	68	9.1
Primary	125	16.8
Intermediate	168	22.6
Secondary	92	12.4
Post-secondary	112	15.1
Tertiary (university etc.)	47	6.3
No information	132	17.7
Family status		
Married	210	28.3
Single	448	60.2
Widowed	24	3.2
No information	62	8.3
I came to Germany with …		
Alone	383	51.5
My partner	67	9.0
My child/children	34	4.6
My partner and my child/children	108	14.5
No information	152	20.4

Note: *n* = sample size (subsample); % = percentage based on total answers.

**Table 2:** Means, standard deviations, standard errors, and Pearson correlations between mental wellbeing, spiritual needs, religious coping and situation appraisal.

	WELL	Spiritual Needs					Religious Coping		APP-N
		SPIR	SPIR-EXIST	SPIR-GEN	SPIR-PEACE	SPIR-REL	RCOPE-P	RCOPE-N	
*M*	3.59	2.62	2.41	2.78	2.72	2.57	2.84	1.71	1.98
*SD*	1.09	0.58	0.78	0.65	0.67	0.79	0.77	0.79	0.97
*SE*	0.04	0.02	0.03	0.02	0.02	0.03	0.03	0.03	0.04
Spiritual Needs									
Total (SPIR)	0.28 **								
Existential (SPIR-EXIST)	0.33 **	0.84 **							
Generativity (SPIR-GEN)	0.17 **	0.84 **	0.63 **						
Inner Peace (SPIR-PEACE)	0.08 *	0.66 **	0.33 **	0.50 **					
Religious (SPIR-REL)	0.27 **	0.83 **	0.67 **	0.58 **	0.32 **				
Religious Coping									
Positive (RCOPE-P)	0.06	0.32 **	0.14 **	0.30**	0.24 **	0.33 **			
Negative (RCOPE-N)	−0.16 **	0.21 **	0.39 **	0.07	−0.16**	0.30 **	0.04		
Situation Appraisal									
Negative (APP-N)	−0.05	0.11 **	0.29 **	−0.01	−0.11**	0.15 **	−0.22 **	0.44 **	

Note: WELL = mental wellbeing, * *p* < 0.05. ** *p* < 0.01.

**Table 3:** Model coefficients for the mediation model with serial and parallel mediation properties.

Consequent		Antecedent				ANOVA Regression Result
		X (APP-N)	M_1_ (SPIR)	M_2_ (RCOPE-P)	M_3_ (RCOPE-N)	
M_1_ (SPIR)	B	0.06				*R*^2^ = 0.01 *F*(1, 742) = 8.51, *p* < 0.01
	SE B	0.02				
	β	0.11 **				
M_2_ (RCOPE-P)	B	−0.18	0.39			*R*^2^ = 0.12 *F*(2, 742) = 52.61, *p* < 0.001
	SE B	0.03	0.05			
	β	−0.23 **	0.30 **			
M_3_ (RCOPE-N)	B	0.36	0.23			*R*^2^ = 0.24 *F*(2, 742) = 117.59, *p* < 0.001
	SE B	0.03	0.04			
	β	0.44 **	0.17 **			
Y (WELL)	B	−0.18	0.45	−0.07	−0.25 **	*R*^2^ = 0.09 *F*(4, 742) = 18.01, *p* < 0.001
	SE B	0.05	0.07	0.05	0.06	
	β	−0.16 **	0.24 **	−0.05	−0.18 **	

Note: Model coefficients are reported in unstandardized and standardized form. APP-N = negative situation appraisal; SPIR = spiritual needs; RCOPE-P = positive religious coping; RCOPE-N = negative religious coping; WELL = mental wellbeing. ** *p* < 0.01.

**Table 4:** Total, direct and indirect effects in the mediation model with serial and parallel mediation properties.

Effect	B	SE B	β	95% CI		t	*p*
				Lower	Upper		
Specific indirect effect through M_1_ (APP-N → SPIR → WELL)	0.028	0.011	0.025	0.008	0.053		
Specific indirect effect through M_2_ (APP-N → RCOPE-P → WELL)	0.013	0.010	0.012	−0.007	0.035		
Specific indirect effect through M_3_ (APP-N → RCOPE-N → WELL)	−0.089	0.023	−0.079	−0.044	−0.134		
Specific indirect serial effect through M_1_ and M_2_ (APP-N → SPIR → RCOPE-P → WELL)	−0.002	0.002	−0.002	−0.006	0.001		
Specific indirect serial effect through M_1_ and M_3_ (APP-N → SPIR → RCOPE-N → WELL)	−0.004	0.002	−0.003	−0.001	−0.008		
Direct effect of APP-N on WELL (*c’*, controlled for mediators)	−0.176	0.046	−0.157	−0.266	−0.086	−3.83	<0.001
Total effect of APP-N on WELL (*c*, direct and indirect effects)	−0.044	0.041	−0.039	−0.124	0.037	−1.06	0.289

Note: Model coefficients are reported in unstandardized and standardized form. Indirect effects are based on 10,000 samples with replacement to produce bias corrected bootstrap confidence intervals with 95% level of confidence. APP-N = negative situation appraisal; SPIR = spiritual needs; RCOPE-P = positive religious coping; RCOPE-N = negative religious coping; WELL = mental wellbeing.

## Data Availability

The data presented in this study are available on request from the corresponding author.
